# Revisiting cancer hallmarks: insights from the interplay between oxidative stress and non-coding RNAs

**DOI:** 10.1186/s43556-020-00004-1

**Published:** 2020-08-31

**Authors:** Li Zhou, Zhe Zhang, Zhao Huang, Edouard Nice, Bingwen Zou, Canhua Huang

**Affiliations:** 1 State Key Laboratory of Biotherapy and Cancer Center, West China Hospital, and West China School of Basic Sciences & Forensic Medicine, Sichuan University, and Collaborative Innovation Center for Biotherapy, Chengdu, 610041 P.R. China; 2 Department of Biochemistry and Molecular Biology, Monash University, Clayton, Victoria 3800 Australia; 3 Department of Thoracic Oncology and Department of Radiation Oncology, Cancer Center, West China Hospital, Sichuan University, Chengdu, 610041 P.R. China; 4 School of Basic Medical Sciences, Chengdu University of Traditional Chinese Medicine, Chengdu, 611137 P.R. China

**Keywords:** ncRNAs, Oxidative stress, ROS, Cancer hallmarks

## Abstract

Cancer is one of the most common disease worldwide, with complex changes and certain traits which have been described as “The Hallmarks of Cancer.” Despite increasing studies on in-depth investigation of these hallmarks, the molecular mechanisms associated with tumorigenesis have still not yet been fully defined. Recently, accumulating evidence supports the observation that microRNAs and long noncoding RNAs (lncRNAs), two main classes of noncoding RNAs (ncRNAs), regulate most cancer hallmarks through their binding with DNA, RNA or proteins, or encoding small peptides. Reactive oxygen species (ROS), the byproducts generated during metabolic processes, are known to regulate every step of tumorigenesis by acting as second messengers in cancer cells. The disturbance in ROS homeostasis leads to a specific pathological state termed “oxidative stress”, which plays essential roles in regulation of cancer progression. In addition, the interplay between oxidative stress and ncRNAs is found to regulate the expression of multiple genes and the activation of several signaling pathways involved in cancer hallmarks, revealing a potential mechanistic relationship involving ncRNAs, oxidative stress and cancer. In this review, we provide evidence that shows the essential role of ncRNAs and the interplay between oxidative stress and ncRNAs in regulating cancer hallmarks, which may expand our understanding of ncRNAs in the cancer development from the new perspective.

## Introduction

Cancer initiation and progression are caused by alterations in key processes, which allow cancer cells to acquire specific characteristics summarized by Hanahan and Weinberg as “The Hallmarks of Cancer” [1]. They have proposed eight hallmarks (sustaining proliferative signaling, evading growth suppressors, activating invasion and metastasis, enabling replicative immortality, inducing angiogenesis, resisting cell death, reprogramming energy metabolism, and avoiding immune destruction) and two enabling characteristics (genome instability and mutation and tumor-promoting inflammation) in which successive alterations accumulate in multiple protein-coding and noncoding genes. Indeed, protein-coding genes play central roles in regulation cancer progression, and many studies have been conducted to explore the potential mechanisms [2–5]. However, large-scale genome sequencing has revealed that more than 90% of the human genome is actively transcribed, but less than 2% of the total genome encodes proteins. Thus, non-coding RNAs (ncRNAs) are major component of the human transcriptome and affect normal expression of the genes, including oncogenes and tumor suppressive genes, which make them a new class of targets for drug discovery in cancer.

Reactive oxygen species (ROS) are generally defined as oxygen-containing free radicals with highly reactive properties such as hydroxyl free radicals (HO^•^) and superoxide (O^2•−^) and non-radicals such as hydrogen peroxide (H_2_O_2_) [6]. Conventional doctrine suggested that ROS were only metabolic waste, which were harmful to nucleic acids, lipids, and proteins [7]. In fact, ROS play dual roles in biological events according to their cellular level. Low or moderate concentration of ROS act as important second messengers to regulate multifarious signaling pathways involved in cell proliferation, apoptosis, migration, DNA damage, differentiation and chemoresistance [8]. In contrast, high concentration of ROS cause damage to cellular macromolecules such as DNA, lipid, and proteins, which leads to induction of apoptosis [9]. Indeed, ROS levels are balanced via several detoxification processes regulated by antioxidant enzymes, which is termed “Redox Homeostasis” [10]. The disruption of redox homeostasis is known as “oxidative stress”, which drives key cellular physiological regulatory responses and leads to diverse pathological conditions [11]. Cancer cells are usually in a chronic state of oxidative stress, as evidenced by elevated ROS levels and accompanied down-regulation of cellular antioxidant systems [12]. This form of physiological oxidative stress (eustress) endow cancer cells with a sustained proliferative and aggressive phenotype to promote malignant transformation, whereas excessive oxidative burden (distress) cause damage to cellular macromolecules and are toxic to cancer cells [11]. It is therefore important to investigate the detailed mechanisms underlying oxidative stress-induced cancer progression for better cancer management.

In this review, we introduce recent advances in the understanding of ncRNAs involved in cancer hallmarks, particularly focusing on their interplay with ROS, which will contribute to a better understanding of cancer progression and may benefit the development of novel strategies for cancer treatment.

## General characterization and functions of ncRNA

ENCODE indicates that, although much of the human genome is transcribed into RNAs, the majority of these do not code for proteins [13]. These ncRNAs are grouped into two major classes based on the transcript size: small ncRNAs and long ncRNAs (lncRNAs) (Table 1) [14]. The size of small ncRNA is usually less than 200 nucleotides (nt). These are divided into microRNA (miRNA), PIWI-interacting RNA (piRNA), small nucleolar RNA (snoRNA), small nuclear RNA (snRNA), small interfering RNA (siRNA), and the new class of tRNA-derived fragments (tRF) [15, 16]. miRNA is a class of small non-coding RNA whose length is about 19–24 nt. They regulate gene expression processes by binding to miRNA response elements (MREs) in RNA sequences and inhibiting subsequent translation by inducing degradation of target RNA transcripts [17]. piRNAs are small RNAs 24–32 nt in length which are derived from repeated sequences within the genome. They are identified as endogenous siRNAs and regulate germ cell development, stem cell self-renewal and the silencing of transposons [18]. The snoRNAs comprise a class of nucleolus-enriched ncRNAs with conserved stem motifs that act as guides to induce chemical modification and maturation of other non-coding RNA, such as rRNA [19]. snoRNAs are also further processed into miRNAs that target cellular mRNAs. The tRNA-derived fragments are a number of sequencing reads which map to RNA fragments derived from the cleavage of tRNA transcripts, and have potential implication in gene suppression as well as many other cell functions which are summarized in these reviews [20, 21].

**Table 1:** General characterization and function of main ncRNAs

	ncRNA types	Length	Cellular distribution	Molecular functions
Small ncRNAs	miRNA	19–24 nts	Nucleus and cytoplasm	Suppressing gene translation
	piRNA	24–32 nts	Cytoplasm	Suppressing gene translation and regulating transposons
	snoRNA	60–300 nts	Nucleus and cytoplasm	Implicating in the chemical modification of rRNA
	snRNA	100–200 nts	Nucleus and cytoplasm	Component of spliceosome
	siRNA	20–30 nts	Cytoplasm	Repression gene translation
	tRF	14–40 nts	Nucleus and cytoplasm	Suppressing gene expression, regulating cell proliferation and RNA processing
Long ncRNAs	Signaling lncRNA	> 200 nts	Nucleus and cytoplasm	Regulating signaling cascade
	Decoy lncRNA	> 200 nts	Nucleus and cytoplasm	Regulating gene expression
	Guide lncRNA	> 200 nts	Nucleus and cytoplasm	Guiding protein complexes to target genes
	Scaffold lncRNAs	> 200 nts	Nucleus and cytoplasm	Regulating gene expression and chromosomal dynamics
	CircRNA	200–800 nts	Nucleus and cytoplasm	miRNA sponge, regulating gene transcription

In contrast, long ncRNA is usually more than 200 nt length and is classified into different categories according to their localization in genome, modes of action, and function [22]. On the basis of their localization in genome, lncRNAs are divided into: intronic lncRNAs generated from the region in introns of protein-coding genes; intergenic lncRNAs (lincRNA) generated from the region between two protein-coding genes; enhancer lncRNAs (elncRNA) generated from the regions in enhancer; bidirectional lncRNAs generated from the region in the vicinity of a coding transcript of the opposite strand; sense-overlapping lncRNAs overlapped with several introns and exons of divese protein-coding genes in DNA sense strand; antisense transcripts generated from the DNA antisense strand [23].

According to their diverse functions, lncRNAs are classified as signaling, decoy, guide, and scaffold lncRNAs [24]. Signaling lncRNAs are involved in specific signaling pathways and their expression presents an active signaling event, with either direct or indirect roles. Decoy lncRNAs regulate gene expression by serving as a decoy to prevent access of transcription factors to chromatin or to competitively bind to miRNA. For example, lncRNA GAS5 (growth arrest specific 5) was found to directly interact with the WW domain of YAP to facilitate translocation of endogenous YAP from the nucleus to the cytoplasm, thus promoting phosphorylation and subsequently ubiquitin-mediated degradation of YAP to inhibit colorectal cancer (CRC) progression in vitro and in vivo [25]. Guide lncRNAs bind to the regulatory or enzymatically active protein complexes and recruit them to target genes. For instance, the p53-regulated long noncoding RNA lincRNA-p21 has been reported to act in concert with hnRNP-K as a coactivator for p53-dependent p21 transcription, thus promoting Polycomb target gene expression and enforcing the G1/S checkpoint [26]. Scaffold lncRNAs function as scaffolds to form various protein complexes, which affect gene expression and chromosomal dynamics. For example, metastasis associated with lung adenocarcinoma transcript-1 (MALAT1), a functional long non-coding RNA highly expressed in colorectal cancer cells, promotes cell proliferation and migration by binding to SFPQ, thus releasing PTBP2 from the SFPQ/PTBP2 complex [27].

In addition to these linear ncRNAs, a new type of circular ncRNA (circRNA) has recently been identified. The structure of circRNAs differs from other linear lncRNAs in which their 3′ and 5′ ends are not free but covalently joined. CircRNAs are mainly arised from the exons of protein-coding genes, as well as the intronic, intergenic, UTR-regions, ncRNA loci and locations antisense to known transcripts [28, 29]. Because of their distinct structure, circRNAs are resistant to nucleases and have a relatively long half-life, making them relatively easy to detect in tissues, serum, and urine as potential biomarkers for human cancer [24]. Indeed, circRNAs are implicated in a variety of cancers (including gastric cancer, hepatocellular cancer, bladder cancer, and esophageal cancer, and others) by acting as competitive endogenous RNAs (ceRNAs), which regulate gene expression via the competitive binding of miRNA. An example of this is ciRS-7, which acts as a sponge for miR-7 to promote colorectal cancer progression by releasing the repression of oncogenes such as YY1 by tumor suppressor miR-7 [30].

In summary, ncRNAs, particularly miRNAs and lncRNAs, are emerging as a novel class of regulators associated with modulation of cellular biological processes and are closely related to tumorigenesis, which allows them to serve as potential diagnosis and prognosis biomarkers for cancer therapy.

## Oxidative stress in cancer progression

ROS are broadly defined as oxygen-containing species with reactive properties, including hydroxyl free radicals (HO^•^), non-radical molecules (hydrogen peroxide, H_2_O_2_, etc.) and superoxide (O^2•−^). These molecules are the principal byproducts of various metabolic reactions occurring in the mitochondria, peroxisomes and the endoplasmic reticulum (ER) [31]. ROS have been widely accepted as second messengers which are involved in many different biological events. Low to moderate levels of ROS act as signaling transduction molecules and promote cell proliferation and differentiation, as well as stress tolerance. However, superfluous ROS lead to cell death through irreversible damage to DNA, proteins or lipids. Therefore, tightly regulated ROS generation and detoxification are crucial for sustaining cellular physiological processes.

Elevated ROS levels, due to imbalance of ROS generation or elimination, is termed oxidative stress and is one of the hallmarks of cancer. Oxidative stress has been proposed to orchestrate tumorigenesis and tumor progression through direct or indirect mechanisms. The first link between ROS and tumorigenesis was identified in 1981, when insulin was found to promote the accumulation of H_2_O_2_ thus potentiating tumor cell proliferation [32]. The conventional thought is that excessive ROS promote the mutation of DNAs and lead to irreversible oxidation of proteins and lipids, which probably activate oncogenic signaling pathways and facilitate tumorigenesis. However, recent evidence suggests that low or moderate levels of ROS induce reversible oxidative modification of proteins which affects their function, regulating tumor apoptosis, proliferation, invasion, inflammation and drug resistance. Mechanistically, proteins possess redox-sensitive cysteine residues that can be oxidized by ROS. Several oxidative modification patterns have been reported, including disulfide bonds, sulfenylation, sulfinylation, sulfonylation and S-glutathionylation. Because of these redox modifications, ROS alter the biological functions of redox-sensitive proteins involved in most hallmarks of cancer (e.g. pyruvate kinase M2 (PKM2) in regulating metabolism reprogramming, receptor tyrosine kinases (RTK) in sustaining proliferative signaling, p53 in evading growth suppression), thereby regulating cancer cell progression [33–35].

## The intrinsic links between ROS and ncRNAs with cancer hallmarks

### ROS and ncRNAs in sustaining proliferative signaling

An initial key hallmarks of cancer is constitutive cell proliferation and avoidance of growth arrest. In normal cells, the cell cycle progress and subsequent proliferation are tightly controlled to avoid aberrant cell growth and malignant transformation. However, cancer cells maintain sustained proliferation via amplification or mutation of certain genes, especially those encoding for kinases and kinase receptors, which have attracted a lot of attention as potential therapeutic targets.

#### Cyclin-dependent kinases (CDKs): the direct regulators of cell proliferation regulated by ROS and ncRNAs

The proliferation of cancer cells is precisely controlled by entry of specific phases of the cell cycle which are regulated by cyclins, CDKs, and CDK inhibitors (CDKIs). The CDKs, particularly CDK1, CDK2, and CDK4/6, are activated via binding to their selected cyclins: cyclin B, cyclin E and cyclin D, respectively, to form functional complexes which give access to the entry of the cell cycle [36]. CDKIs, such as p16, p21, and p27, act as negative regulators of activated CDKs by specifically binding to their target cyclin-CDK complexes to block the cell cycle progression [37]. After these regulators complete their functions in controlling cell cycle, they are commonly ubiquitylated by specific E3 ligases and degraded via the ubiquitin-proteasome pathway [38].

In addition to post-translational regulation, these cell cycle regulators are also modulated by translational regulation in which ncRNAs play a fundamental role. Previous studies have demonstrated that *CDK4* and *CDK6* are directly regulated by a variety of ncRNAs, some of which are closely related to cellular ROS. For instance, it is well-known that ionizing radiation (IR) induces ROS generation in cancer cells and multiple ncRNAs may be dysregulated under this stress condition [39]. The miRNA let-7 family, a well-established tumor suppressive ncRNA, has been found to be downregulated under ionizing radiation-induced oxidative stress, leading to the expression of CDK6 and subsequently promoting cell cycle progression of melanoma cells [40]. Likewise, 5-aminolevulinic acid-mediated sonodynamic therapy (ALA-SDT) treated melanoma cells showed increased intracellular ROS levels and miR-34a expression, which acted synergistically to inhibit the expression of pro-proliferative factors Cyclin D1 and CDK6 to suppress cell cycle progression [41]. Two other tumor suppressors, miR-15a and miR-16 were downregulated in several cancer types [42, 43]. Overexpression of miR-15a and miR-16 mediated production of mitochondrial ROS and inhibition of cell cycle regulators including cyclin D1, cyclin E1, cyclin D3 and CDK6, thereby inducing cell cycle arrest at the G1 phase [44, 45]. In addition, silencing of miR-21 in A549 human lung cancer cells increased oxidative damage and the cell cycle was blocked at the G0/G1 phase by downregulation of CDK1, thus reversing multidrug resistance of lung cancer cells [46]. In prostate cancer cells, ionizing radiation upregulated the expression of miR-17-3p, which led to cellular ROS accumulation and cell cycle arrest by targeting manganese superoxide dismutase (MnSOD) and cyclin D1, respectively [47]. In colon cancer cells, curcuminoid treatment induced ROS production which disrupted the miR-27a/Sp/cyclin B/cdc2 axis and inhibited subsequent G2-M transition, leading to enhanced growth inhibition of 5-FU [48, 49].

**Fig. 1:**
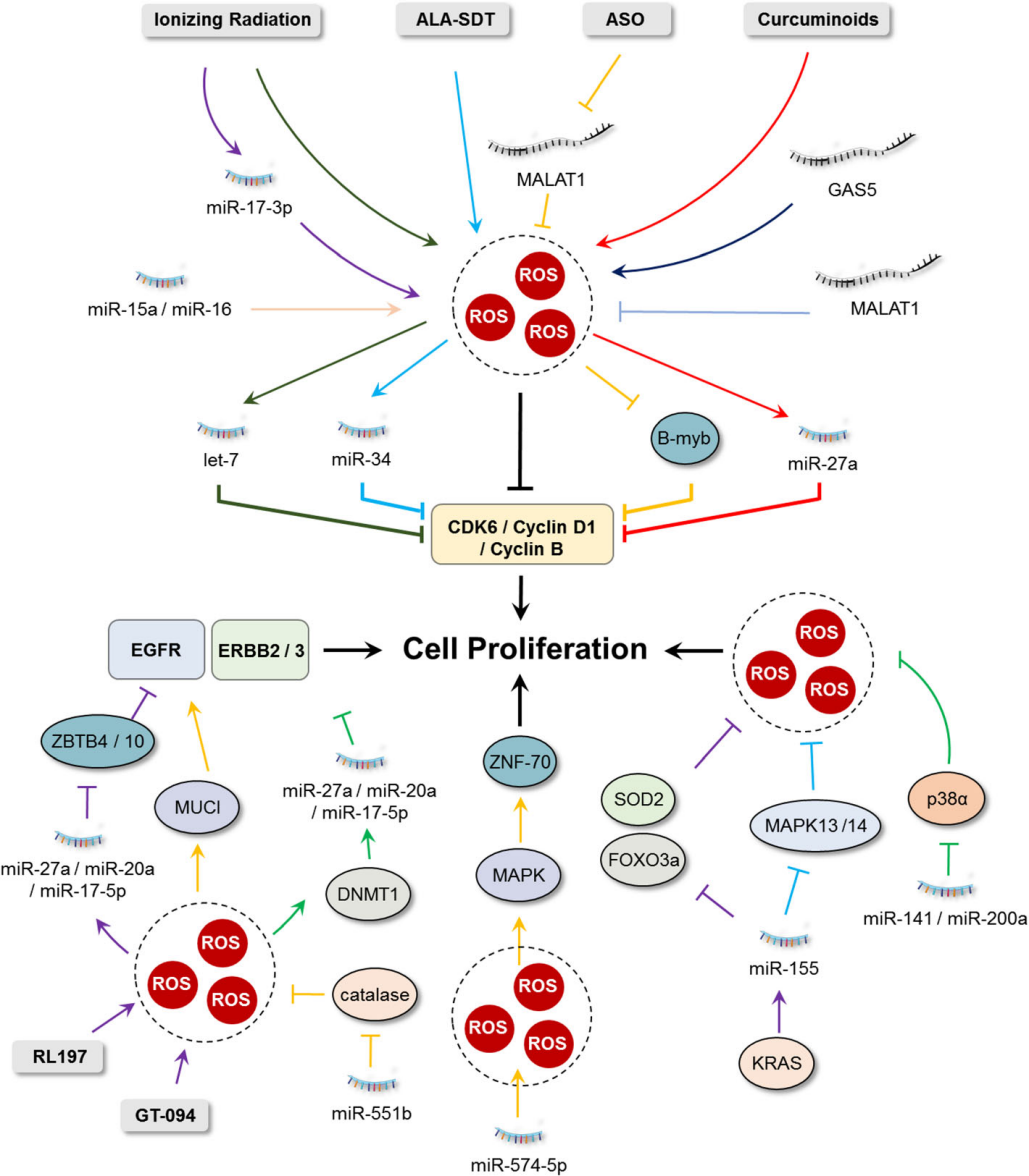
Interplay between ROS and ncRNAs in regulating cancer cell proliferation. Cyclins and cyclin-dependent kinases (CDKs), especially CDK4/6 and Cyclin B/D1, are directly regulated by a variety of ncRNAs, some of which are closely related to cellular ROS. The important roles of interactions between RTKs (including EGFR and MAPK), ncRNAs and ROS in cancer cell proliferation are presented

Except for miRNAs, lncRNAs also participate in regulation of CDKs in cancer cells. Recent study has found that the long noncoding RNA, growth arrest-specific transcript 5 (GAS5) was downregulated in melanoma cells. The reduced expression of lncRNA GAS5 contributed to redox balance and cell cycle progression through increasing expression of Cyclin D1, CDK4, and NOX4, suggesting downregulated GAS5 and increased ROS levels as promising diagnosis or prognosis biomarker for malignant melanoma [50]. In Hepatitis C virus-related hepatocellular carcinoma (HCC), microarray analysis has found that lncRNA LINC01419 and AF070632 are mostly involved with cell cycle progression and oxidation-reduction, although the underlying mechanism remains unclear [51]. MALAT1, an evolutionarily conserved lncRNA that regulates mRNA splicing [52], is upregulated in several types of human cancers and is involved in cancer cell proliferation. Targeting MALAT1 by a novel LNA gapmeR antisense oligonucleotide (ASO) resulted in increased ROS levels and decreased expression of B-Myb, which was responsible for the expression of mitotic proteins such as cyclin B1, CDK1 [53, 54]. These above findings indicate that cellular ROS and ncRNAs are tightly regulated by each other and both directly modulate expression of CDKs either positively or negatively in a context dependent manner, thus supporting the cancer hallmark “sustaining proliferation” (Fig. 1).

#### RTKs are the upstream growth sensors regulated by ROS and ncRNAs

The proliferation of cancer cells, regulated by cyclins and CDKs, also requires stimulation of upstream signals that are sensed by RTKs after growth factor treatment [55]. Dysregulation of RTKs (either gene amplification or somatic mutations) that results in their constitutive activation and oncogenic properties, has been reported in various tumor types, contributing to tumor growth [56]. Accumulating evidence supports the important role of interactions between RTKs, ncRNAs and ROS in cancer cell proliferation (Fig. 1).

The epidermal growth factor receptor (EGFR) is one of the most studied RTKs. It is activated by binding of its ligand, the epidermal growth factor (EGF), resulting in activation of signaling pathways promoting proliferation [57]. Studies have shown that ncRNAs, along with EGFR and cellular ROS, display crucial roles in the progression of cancer. Curcumin is the major active ingredient in turmeric which inhibits growth of several cancer cell lines by downregulating specificity protein (Sp) transcription factor Sp1 and its target genes [58, 59]. Gandhy and colleagues found that curcumin and its synthetic analog RL197 induced ROS accumulation in colon cancer cells, which decreased expression of Sp1, Sp3, Sp4 and Sp-regulated genes, including EGFR [60]. They further revealed that curcumin−/RL197-induced suppression of Sp was dependent on ROS and the subsequent induction of the Sp repressors ZBTB10 and ZBTB4 regulated by decreased miR-27a, miR-20a and miR-17-5p [60]. Likewise, the miR-27a/ZBTB10/Sp/EGFR axis was also observed in colon cancer cells after treatment with ethyl 2-((2,3-bis(nitrooxy)propyl)disulfanyl)benzoate (GT-094, a novel nitric oxide chimera that induces ROS production) [61]. In lung cancer cells, increased miR-551b expression reduced the expression of catalase and potentiated cellular ROS levels and MUC1 expression. Upregulation of MUC1 promoted EGFR-mediated activation of Akt/c-FLIP/COX-2 to support cell survival, thereby protecting cancer cells from damage caused by anticancer agents [62]. In another study, high ROS generation in human ovarian cancer cells inhibited the expression of miR-199a and miR-125b by increasing promoter methylation mediated by DNA methyltransferase 1. Decreased expression of miR-199a and miR-125b thus activated ERBB2 and ERBB3 to promote tumor progression [63].

The mitogen-activated protein kinases (MAPK)/extracellular signal related kinases MEK1/2 are another important RTKs involved in cell proliferation. Among the complex family members, RAS/RAF/MAPK is one of the major pathways for targeted cancer therapy and several ncRNAs are known to regulate this signaling cascade [64]. The three canonical RAS family genes (K-RAS, N-RAS and H-RAS) are commonly mutated and hyperactivated in several cancer types, among which the K-RAS is one of the drivers for pancreatic adenocarcinomas [65]. A miRNA array-based study has been conducted to identify dysregulated miRNAs under K-RAS activation. The authors found that miR-155 was the most upregulated miRNA and caused inhibition of FOXO3a and decrease of major antioxidants (including SOD2 and catalase), thus enhancing pancreatic cell proliferation induced by ROS generation [66]. In addition, miR-155 also functions in regulation of glioblastoma cell proliferation. Knockdown of miR-155 sensitizes glioma cells to chemotherapy with temozolomide by releasing the expression of MAPK13 (also known as p38 MAPKδ) and MAPK14 (also known as p38 MAPKα), two tumor suppressor genes that lower the accumulation of ROS and induce apoptosis [67]. In esophageal squamous cell carcinoma (ESCC), miR-574-5p was upregulated both in vitro and in vivo which correlated with ZNF70 expression. Further study suggested that upregulation of miR-574-5p promoted mitochondrial ROS generation and MAPK pathways, which was closely related to ZNF70-regulated cell proliferation [68]. Another study has shown the interaction between oxidative stress and the miR-200 family in ovarian cancer progression. High expression of miR-141 and miR-200a targets p38α to modulate the oxidative stress response, thereby promoting tumorigenesis and chemoresistance. This crucial role of miR-200a in stress response may serve as a predictive marker for clinical outcome in ovarian cancer [69].

### ROS and ncRNAs in evading growth suppression

**Fig. 2:**
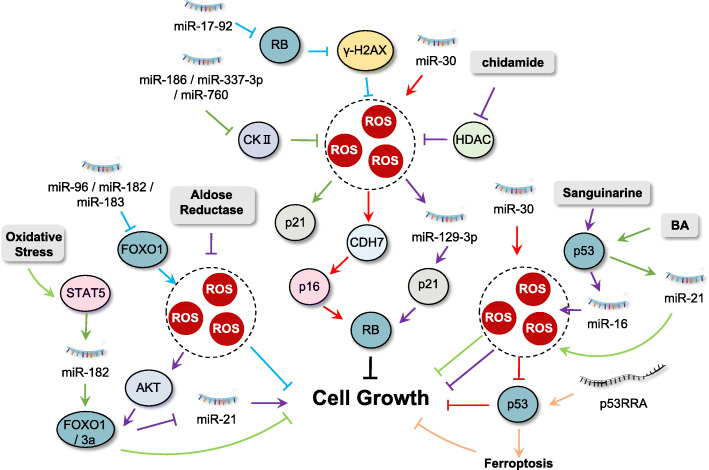
Interplay between ROS and ncRNAs in evading growth suppression. Tumor suppressor genes, including retinoblastoma (RB) protein, p53 and forkhead box O (FOXO) transcription factors are involved in suppression of cell proliferation, regulated by the interplay between ROS and ncRNAs

In addition to constitutive cell proliferation, cancer cells need to undergo certain cellular events to avoid growth suppression caused by tumor suppressors. Several tumor suppressor genes, including retinoblastoma (RB) protein, p53 and forkhead box O (FOXO) transcription factors, are involved in suppression of cell proliferation or in guiding the cells to an irreversible growth arrest termed cellular senescence. ROS in cancer cells have been reported to interact with ncRNAs for positive or negative regulation of these growth suppressors, thereby modulating the evasion of cancer cells (Fig. 2).

#### RB protein-a cell cycle gatekeeper regulated by ROS and ncRNAs

*RB1* gene is a tumor suppressor gene whose inactivation is responsible for one of the important childhood malignancies, retinoblastoma (Rb). It exhibits tumor suppressor function by restricting the entry from G1 to S phase of the cell cycle, acting as a gatekeeper that controls cell proliferation and quiescence [70]. Multiple evidence has indicated that deregulation of various ncRNAs is involved in RB protein functions. For example, knockdown of RB in NSCLC cells induced gamma-H2AX foci formation, resulting in ROS generation and growth inhibition, which was attenuated by overexpression of miR-17-92 [71]. In human triple-negative breast cancer (TNBC) cells, the expression of human long noncoding RNA LINK-A enhanced K48-polyubiquitination-mediated degradation of the antigen peptide-loading complex (PLC) and intrinsic tumor suppressors RB [72]. In NSCLC H1355 and A549 cells, treatment with chidamide, a histone deacetylase inhibitor (HDACi), induced ROS accumulation and G1 arrest through the regulation of p21 and pRB by miR-129-3p [73]. The tumor suppressor p16^INK4A^ is an essential regulator of RB activity and plays essential roles in oncogene-induced senescence [74]. Under ROS condition, p16^INK4A^ gets activated and functions by binding to and inhibiting CDK4/CDK6. miR-30, a miRNA frequently overexpressed in human cancers, has been found to disrupt senescence and promote cancer by suppressing 2 targets, CHD7 (a transcriptional coactivator essential for induction of p16^INK4A^) and TNRC6A (a miRNA machinery component required for repairing oxidative stress-induced DNA damage) [75]. Downregulation of protein kinase CKII induces cellular senescence in human colon cancer cells. Four miRNAs (miR-186, miR-216b, miR-337-3p, and miR-760) were found to degrade CKIIα mRNA by targeting its 3′ untranslated regions (UTRs), thereby increasing senescence-associated β-galactosidase (SA-β-gal) staining, p21^Cip1/WAF1^ expression and ROS production [76].

#### FOXOs: the major modulators of cell fate regulated by ROS and ncRNAs

Transcription factors of FOXO families are major regulators of cell growth and death and cellular redox homeostasis [77]. FOXOs display tumor-suppressive functions by mediating the transcription of a plethora of target genes, including *p16*^*INK4A*^, *p19*^*INK4D*^, *p21*^*CIP1/WAF1*^, and *p27*^*KIP1*^ CKIs, keeping cells in a quiescent state in the absence of growth factor stimulation [78]. Many ncRNAs have been identified as regulators of *FOXO* expression. For instance, miR-27a and miR-96 were identified as regulators of FOXO1 expression together with miR-182, and were highly expressed in MCF-7 and MDA-MB-231 breast cancer cells, causing down-regulation of FOXO1 protein levels which contributed to maintenance of a proliferative state while impairing apoptotic responses [79]. In glioma cells, overexpression of miR-96, miR-182 and miR-183 led to a decreased FOXO1 expression and increased cell proliferation, which was accompanied by lower ROS production although FOXOs proteins are usually supposed to be involved in cellular antioxidant response [80]. In addition, oxidative stress inhibits STAT5-mediated miR-182 expression, thereby releasing FOXO1 to suppress growth of human SK-N-MC neuroblastoma cells [81]. In human colon cancer cells, inhibition of aldose reductase significantly increased PTEN and FOXO3a expression via the growth factor-induced ROS/PI3K/AKT axis, thereby downregulating miR-21 expression and inhibiting colon cancer growth [82]. In another study, using microarrays, the authors identified miR-155 as the most upregulated miRNA after both acute and prolonged activation of K-Ras in a doxycyline-inducible system. Overexpression of miR-155 caused inhibition of FOXO3a and enhanced cell proliferation induced by ROS generation in human pancreatic cancer cells [66]. Additionally, overexpression of miR-155 in NSCLC cells increased cell proliferation through inhibition of FOXO1 and subsequent production of ROS [83].

#### p53: a powerful tumor suppressor regulated by ROS and ncRNAs

During the past decade, mounting evidence has suggested that the interplay between p53, ncRNA, and ROS helps in controlling p53-regulated genes that are directly or indirectly linked to suppression of cancer cell growth. p53, often called “the guardian of the genome” is the most well-known tumor suppressor to date [84]. Under normal conditions, p53 is maintained at a lower level due to rapid protein degradation mediated by MDM2 [85]. However, when cells encounter stress conditions, p53 is stabilized and activated to protect cells from stress-induced damage or the cellular apoptotic process is initiated if the damage is irreversible, thereby preventing malignant transformation [86]. miR-16 is a p53-regulated microRNA and is frequently deleted or downregulated in HCC cells [87]. Treatment with sanguinarine in HCC cells activates miR-16 via increased p53 occupancy on the miR-16 promoter. Upregulated miR-16 inhibited expression of its target genes, including Bcl-2 and cyclin D1, thus inducing p53-dependent cell cycle arrest and ROS-associated apoptosis [88]. In gastric cancer tissues and cell lines, miR-30 is overexpressed. This suppresses mitochondrial dysfunction and apoptotic events by decreasing ROS generation and inhibiting p53 activation [89]. Betulinic acid (BA), a pentacyclic triterpene, has been reported to show anti-cancer activity against HCC. Mechanistic study revealed that p53 was responsible for the anti-cancer activity of BA through upregulation of miR-21 and downregulation of SOD2 expression, resulting in mitochondrial ROS production and apoptosis [90]. In HCC cells, four miRNAs (miR-34a-5p, miR-1915-3p, miR-638, and miR-150-3p) were identified as the oxidative stress-responsive miRNAs, among which miR-34a-5p, miR-1915-3p are regulated by a p53-dependent pathway [91]. Proline oxidase (POX) is a novel mitochondrial tumor suppressor regulated by p53 which inhibits proliferation and induces ROS-dependent apoptosis. It has been reported that miR-23b in renal cancer functions as an oncomiR by direct binding to the POX mRNA 3’UTR region [92].

Apart from miRNAs, lncRNAs also participate in regulation of p53 function. In NSCLC cells, treatment with marine actinomycetes derived 1-hydroxy-1-norresistomycin (HNM) increased ROS generation in mitochondria and upregulated the p53 mediated transcriptional regulation of two lncRNAs (LED and LOC285194), leading to cell cycle arrest and subsequent apoptosis [93]. Ferroptosis is a form of programed cell death regulated by iron-dependent lipid ROS accumulation. The cytosolic lncRNA P53RRA is a tumor suppressor in lung cancer which promotes cell-cycle arrest, apoptosis, and ferroptosis via sequestration of p53 in the nucleus [94]. Arsenic trioxide shows marked anticancer activity in the treatment of hematologic malignancies [95]. A recent study has explored the functions of lncRNAs in the resistance to arsenic trioxide in liver cancer. The results demonstrate that arsenic trioxide treatment promotes the expression of lncRNA ROR and the activation of p53, both of which are regulated by arsenic trioxide induced oxidative stress [96]. LncRNA nuclear enriched abundant transcript 1 (NEAT1) was regulated by p53 in response to DNA damage [97]. EGCG (epigallocatechin-3-gallate), a green tea polyphenol, was found to upregulate lncRNA NEAT1 through ROS generation, which induced copper transporter 1 (CTR1) expression. Enhanced expression of CTR1 increased cisplatin intake, thereby promoting sensitivity to cisplatin in NSCLC cells [98].

Except for p53 itself, many pro- and anti-apoptotic factors (regulated by or which regulate p53) are known to interact with ncRNAs in cancer, of which the B-cell lymphoma 2 (Bcl-2) family is the most prominent. The tumor suppressor miR-34 is a transcriptional target of p53. Capsaicin-induced oxidative damage leads to activation of p53 in NSCLC cells. Upregulated p53 thus enhances expression of miR-34a, which in turn inhibits Bcl-2 expression and promotes cell death [99]. Treatment with docetaxel in prostate cancer cells resulted in upregulation of miR-193a-5p, which in turn promoted HO-1 induced expression of Bcl-2, partly counteracting apoptosis regulated by docetaxel-induced oxidative stress [100]. In addition, overexpression of miR-33a was observed in clinical glioma specimens and cell lines, which negatively regulated the expression of SIRT6 via targeting the 3’UTR mRNA, thereby decreasing ROS production and increasing Bcl-2 expression to inhibit apoptosis [101]. miR-21 was frequently overexpressed in several types of cancer, including lung cancer, liver cancer and breast cancer [102–105]. In human breast cancer MCF-7 cells, miR-21 was down-regulated upon metformin treatment and this impeded ROS production and simultaneously inhibited Bcl-2 expression, thereby inducing apoptotic cell death [103, 106]. Moreover, ROS promote gastric carcinogenesis via upregulation of miR-21, which in turn downregulated the expression of programmed cell death 4 protein (PDCD4), a key regulator responsible for translation of p53 mRNA in gastric cancer cells [107].

### ROS and ncRNAs in resisting cell death

Oncogenic transformation results in higher ROS production in cancer cells. When cancer cells are exposed to high levels of ROS that exceed the clearance rate, ROS will become damaging, and the cellular stress response machinery is activated which may lead to apoptosis. In contrast, cancer cells overcome the induction of apoptosis by either inactivating tumor suppressor genes, most notably p53 as mentioned above, or elevation of the apoptotic threshold by elevating expression of anti-apoptotic proteins like Bcl-2 family members, thereby resisting transformation-induced cell death. Moreover, cancer cells usually undergo mutation or amplification leading to hyperactivation of antioxidant genes, most notably Nrf2, to evaluate the threshold of stress tolerance.

#### Nrf2: an important antioxidant regulator modulated by ROS and ncRNAs

Nrf2, a master regulator of cellular antioxidant response, is commonly expressed in the cytoplasm whose activity is negatively regulated by kelch-like ECH-associated protein 1 (Keap1) [108]. Under oxidative stress conditions, cysteine residues of Keap1 are oxidized which inactivate Keap1 function, thereby resulting in stabilization and subsequent nuclear translocation of Nrf2 [109]. After nuclear translocation, Nrf2 functions as a transcription factor via binding to the antioxidant-response-element (ARE) or electrophile-response element (EpRE), which finally transcribes almost 200 genes responsible for detoxification, antioxidation, and metabolism [110]. NcRNAs has been found to regulate the Nrf2 pathway for cancer management [111]. It has been reported that in human breast cancer cells, miR-28 degrades the Nrf2 mRNA by binding to its 3’UTR, which is independent of Keap1 [112]. Similarly, other miRNAs, including miR-507, − 634, and − 129-5p, are also known to negatively regulate Nrf2-mediated pathways [113]. Cisplatin resistance is a common phenomenon in lung cancer therapy. miR-144-3p prevents cisplatin resistance in lung cancer cells by inhibition of Nrf2 [114]. In addition, miR-200a was downregulated in breast cancer and re-expression of miR-200a released Nrf2 from Keap1 via triggering Keap1 mRNA degradation, leading to nuclear translocation of Nrf2 and subsequent transcription of target genes [115]. miR-125b has been found to upregulate peroxiredoxin-like 2A (PRXL2A), an antioxidant protein commonly upregulated in oral squamous cell carcinoma (OSCC), which inhibits the cellular oxidative damage by positive regulation of the Nrf2 signaling pathway [116]. Treatment with arsenite, a well-documented human lung carcinogen, on human bronchial epithelial (16-HBE) cells resulted in miR-155-mediated Nrf2 inactivation, thus enhancing oxidative damage and cell malignant transformation [117]. In MCF-7 cells, treatment with metformin induced miR-34a expression to downregulate the Sirt1/Nrf2 pathway, resulting in increased susceptibility of cancer cells to oxidative stress and TRAIL-induced apoptosis [118].

The modulatory effect of the Nrf2 signaling pathways on the ncRNAs has also been reported in many studies. HDACis are commonly used in the treatment of cancer, but development of resistance often occurs [119]. In the case of HDACis treatment, Nrf2 signaling pathway was found to activate miR-129-3p to trigger the mTOR pathway, thereby stimulating autophagy for degradation of aged or damaged components and macromolecules to suppress the oxidative stress, leading to chemoresistance of the cancer cells [120]. It has been shown that the Nrf2 signaling pathway inhibits the expression of miR-17-5p to down-regulate FPN1, an iron-exporter protein, which results in the aggregation of iron and ROS. Downregulation of FPN1 contributes to the survival and growth of multiple myeloma [121]. These studies demonstrate that the Nrf2 signaling pathway and miRNAs are regulated by each other, and understanding the relationship between Nrf2 and miRNAs will benefit the delineation of the underlying molecular pathways and the treatment of various cancer.

**Fig. 3:**
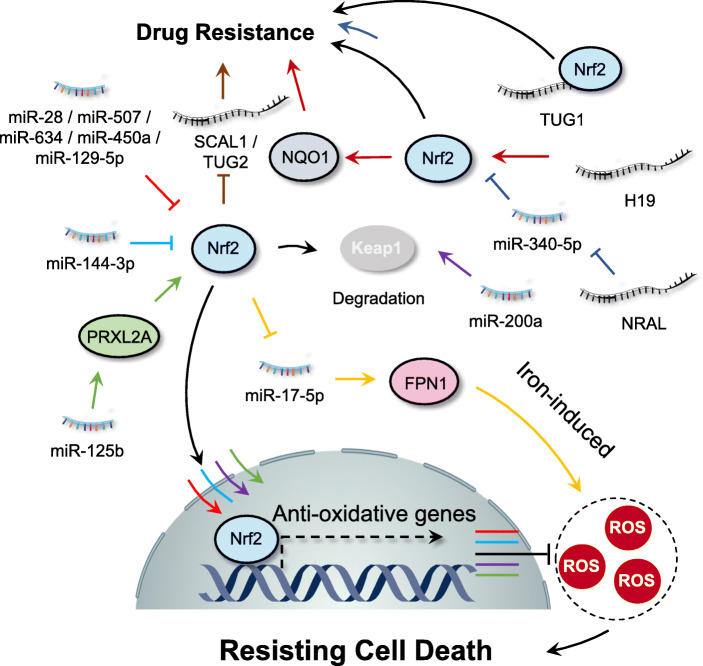
Interplay between ROS and ncRNAs in regulating Nrf-2 pathway. The interaction between ncRNAs and ROS displays a crucial role for both positive or negative regulation of the Nrf-2 pathway which contributing to the cancer hallmark “resisting cell death”

In addition to miRNAs, lncRNAs are also involved in Nrf2-regulated tumorigenesis. LncRNA H19 was found to regulate cisplatin resistance by promoting Nrf2-mediated gene transcription in high-grade serous ovarian cancer [122]. In addition, Nrf2 has been shown to regulate the transcription of smoke- and cancer-associated lncRNA-1 (SCAL1), which decreases oxidative damage in lung cancer cells [123]. In HCC cell lines, it was found that NRAL, a long non-coding RNA, contributed to cisplatin resistance. Further studies revealed that NRAL functioned as a ceRNA to negatively regulate miR-340-5p expression which triggered Nrf2-dependent antioxidant enzymes, suggesting the important role of the NRAL/miR-340-5p/Nrf2 axis in the cisplatin resistance of HCC cells [124, 125]. Another study has found that Nrf2 induces the upregulation of lncRNA taurine-upregulated gene 2 (TUG2), which promotes progression and adriamycin resistance in urothelial carcinoma of the bladder [126]. Conversely, a recent study has suggested that lncRNA TUG1 binds directly to Nrf2 and upregulates its protein expression, thus contributing to cisplatin resistance of ESCC cells [127]. Moreover, lncRNA Keap1 regulation-associated lncRNA (KRAL) functioned as a ceRNA to negatively regulate miR-141 which restored Keap1 expression and inhibited Nrf2 expression, thus reversing 5-FU resistance in HCC cells [128]. In NSCLC cells, miR-335 is the downstream target of lncRNA-XIST and overexpressed lncRNA-XIST increases SOD2 (an important transcriptional target of Nrf2) expression levels by sponging miR-335, thereby decreasing ROS levels and resisting cell death [129]. The above findings therefore suggest that the interaction between ncRNAs, Nrf2 and ROS displays a crucial role for both positive and negative regulation of tumorigenesis (Fig. 3).

#### Cell stemness: key feature of resistant cancer cells which regulated by ROS and ncRNAs

Emerging evidence has shown that a small population of cells with stem cell-like properties, termed cancer stem cells (CSCs), have the capacity to undergo self-renewal which contributes to resistance to conventional therapies [130]. It has been demonstrated that ncRNAs regulate CSC-mediated therapy resistance by modulating the redox state of cancer cells [131]. The stem-like cells in CRC are highly dependent on cellular GSH to maintain ROS levels. Ju and colleagues found that miR-1297 levels were inversely correlated with the expression of xCT, a cystine/glutamate transporter required for GSH synthesis. The interaction of xCT with CD44v, a CSC marker, effectively induced enrichment of CRC stem-like cells, which was associated with cancer aggressiveness and poor therapeutic responses [132]. In another study, the authors found that miR-153 was downregulated and its target gene Nrf-2 upregulated in glioma stem cells (GSCs) when compared to non-GSCs glioma cells. Increased expression of Nrf2 promoted GPX1 expression and subsequently reduced ROS production, leading to radioresistance of GSCs [133]. A further study found that overexpression of miR-153 induced ROS-mediated activation of the p38 MAPK pathway, which significantly reduced stemness and thus enhanced radiosensitivity [133]. In addition, miR-128a promoted ROS production via specific inhibition of the Bmi-1 oncogene, which increased the radiosensitivity of medulloblastoma stem cells [134]. Moreover, Sun and colleagues found that miR-223 expression was downregulated in TNBC stem cells (TNBCSCs) and overexpression of miR-223 resensitized TNBCSCs to TRAIL-induced apoptosis through the mitochondria/ROS pathway [135]. Another study found that the expression of miR-125a was decreased in laryngeal carcinoma tissues and laryngeal cancer stem cells (Hep-2-CSCs). Overexpression of miR-125a reversed cisplatin resistance in Hep-2-CSCs by targeting Hematopoietic cell-specific protein 1-associated protein X-1 (HAX-1, an anti-apoptotic protein associated with mitochondria ROS production) [136].

H19, a well-characterized lncRNA known for its role in embryonic development, was shown to be upregulated in HCC along with ERK/MAPK signaling, which was responsible for the chemoresistance of CD133^+^ stem cells. Inhibition of H19 downregulated ERK signaling and promoted ROS production, resulting in reversed chemoresistance of CD133^+^ cells [137]. Together, these results suggest that dysregulation of cellular redox balance may serve as a major factor for chemo/radio-resistance of CSCs. NcRNAs-mediated redox state alteration may act as a novel strategy to decrease CSCs population, thus overcoming therapy resistance.

#### Autophagy: a key pro-survival process regulated by ROS and ncRNAs

Autophagy, a conserved intracellular self-digestion process responsible for the recycling of damaged proteins or organelles, has been reported to mediate resistance to chemo/radiotherapy-induced cell death [138]. The interaction between ncRNAs and cellular ROS regulates the autophagy process that displays important functions in modulating therapy resistance. For example, miR-17-5p is downregulated in paclitaxel-resistant lung cancer cells. Overexpression of miR-17-5p improved sensitivity of these cells to paclitaxel via targeting Beclin 1 and the subsequent autophagy process, which was accompanied by ROS-mediated apoptosis [139]. In glioma cells, inhibition of miR-21 was found to enhance tamoxifen-induced autophagic cell death which was accompanied by oxidative stress induction and JNK activation, thereby reversing tamoxifen resistance in human glioma cells [140]. Environmental airborne particulate matter PM_2.5_ induced ROS production that was responsible for increased expression of lncRNA loc146880 and further activation of autophagy. Further study found a positive correlation between loc146880 expression and LC3B levels in tumor tissues of lung cancer patients, indicating an oncogenic role of PM_2.5_-induced autophagy [141]. In addition, lncRNA SCAMP1 activates ZEB1/JUN and autophagy to promote pediatric renal cell carcinoma under oxidative stress via targeting miR-429 [142]. A recent study has found that miR-27a reversed chemoresistance of breast cancer cells to doxorubicin by disrupting ROS homeostasis and impairment of autophagy [143]. Treatment of sodium butyrate (NaB, a HDACi) in bladder cancer cells induced AMPK/mTOR pathway-mediated autophagy and ROS overproduction via the miR-139-5p/Bmi-1 axis [144]. miR-30a has also been reported to enhance the chemosensitivity of glioblastoma cells to temozolomide via targeting Beclin-1 and autophagy [145].

### ROS and ncRNAs in deregulating cellular energetics

**Fig. 4:**
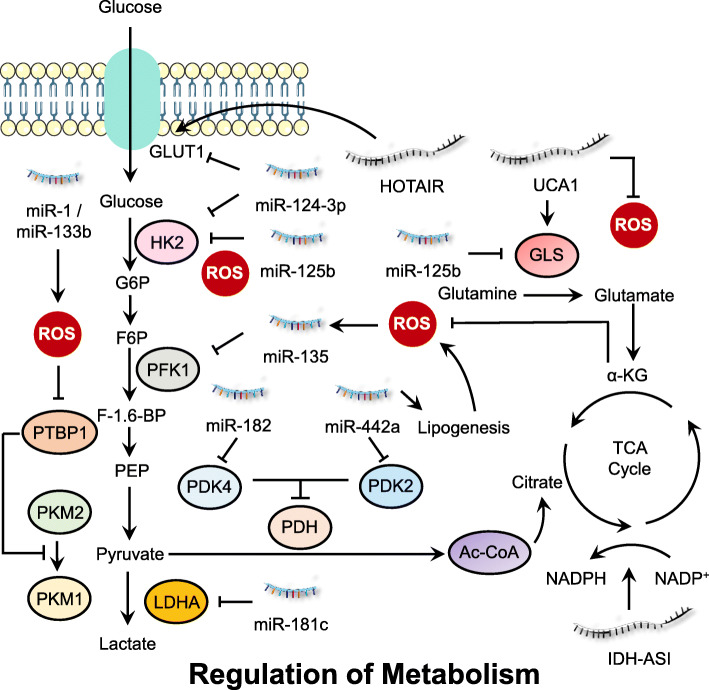
Interplay between ROS and ncRNAs in regulating cell metabolism. Cancer cell metabolism (including glucose, amino acid and lipid metabolism) are tightly regulated by the interaction between ROS and ncRNAs, which generate energy or metabolic intermediates to support cancer cell growth

Metabolic reprogramming is one of the key hallmarks of malignant tumors [146]. Unlike normal cells, cancer cells undergo glycolysis to produce lactic acid even in the presence of oxygen. This was first discovered by Warburg in the 1920s and termed “the Warburg effect” [147]. Since then, many studies have been conducted to thoroughly investigate the possible relationships between metabolism and cancer progression. Metabolism is a central process for cellular redox homeostasis and related signaling, as mitochondria oxidative phosphorylation (OXPHOS) is one of the major sources of ROS [148]. Moreover, cellular reductants (such as NADPH and GSH) are synthesized by metabolic intermediates generated in multiple metabolic processes [149]. Besides ROS, ncRNAs are also involved in regulation of metabolic reprogramming, suggesting a possible role for the interplay between ncRNAs and ROS in modulation of multiple metabolic process (Fig. 4).

#### Regulation of glucose metabolism by ROS and ncRNAs

NcRNAs elaborately regulate cancer-associated glycolytic pathways by modulating expression of specific metabolic enzymes or transcription factors responsible for master regulation of cell metabolism. In pancreatic ductal adenocarcinoma (PDAC) cells, it was observed that glutamine deprivation resulted in accumulation of ROS in MIA-PaCa-2 cells, which promoted miR-135 expression. ROS-mediated upregulation of miR-135 targeted phosphofructokinase 1 (PFK1) and suppressed aerobic glycolysis, thereby promoting the utilization of glucose to support the tricarboxylic acid (TCA) cycle [150]. Another study found that overexpression of miR-422a in gastric cancer cells repressed its target pyruvate dehydrogenase kinase 2 (PDK2) to restore activity of the pyruvate dehydrogenase (PDH), which shifted the energy metabolism from aerobic glycolysis to oxidative phosphorylation [151]. The miR-422a/PDK2 axis also influenced de novo lipogenesis in cancer cells, which subsequently affected ROS generation and RB phosphorylation, resulting in cell cycle arrest at the G1 phase [151].

Accumulation of ROS due to glucose deprivation leads to decreased HDACs activity, and particularly reduced levels of HDAC2, which results in the increased acetylation of miR-466 h-5p promoter region and upregulation of this miRNA [152]. In hypoxic breast cancer cells, oxidative stress-induced overexpression of miR-181c blocked HIF-1α accumulation and diminished hypoxia-inducible levels of glycolysis enzymes, including glycolysis-associated glucose transporter-1 (GLUT1), hexokinase 2 (HK2), PDK1, and lactate dehydrogenase A (LDHA) [153]. Colorectal cancer cells show reduced expression of miR-1 and miR-133b. Ectopic expression of these miRNAs results in ROS generation to silence polypyrimidine tract-binding protein 1 (PTBP1), which converts active PKM2 to inactive PKM1, thus inducing growth suppression and autophagic cell death [154]. Likewise, miR-1 and miR-206 are transcriptional targets of Nrf2 in lung cancer cells. Sustained activation of Nrf2 signaling attenuated miR-1 and miR-206 expression which directed carbon flux toward the pentose phosphate pathway (PPP) and the TCA cycle, contributing to cellular redox homeostasis by enhancing NADPH production [155]. This finding represents a novel link between miRNA, ROS and glucose metabolism in cancer cells.

miR-199a-3p exhibited inhibitory effects on lactic acid production, glucose intake and ROS levels in testicular cancer Ntera-2 cells. Further study found that transcription factor Sp1 was the direct target of miR-199a-3p, which decreased LDHA protein expression [156]. Ectopic expression of miR-143 in renal cell carcinoma resulted in the perturbation of glucose metabolism by negatively modulating the expression of GLUT1 and the PTBP1/PKMs axis, which shifted glycolysis to oxidative phosphorylation and induced autophagy through increasing ROS levels [157]. Using microRNA profiling, Tang and coworkers found that miR-320a inhibited oxidative stress-induced PFK (a rate-limiting glycolytic enzyme) expression in lung cancer cells [158]. Treatment with astragalin, a bioactive component of medicinal plants such as *Rosa agrestis*, resulted in increased miR-125b which repressed HK2 expression and directed glycolysis to oxidative phosphorylation, leading to ROS accumulation and growth suppression of HCC cells [159]. In human lung adenocarcinomas, miR-182 negatively regulates PDK4 and promotes de novo lipogenesis of cancer cells, which is accompanied by increased ROS level and JNK activation [160]. TNBC and metastatic melanoma cell lines show overexpression of let-7a which represses cell proliferation, increases ROS, and downregulates proteins involved in glucose metabolism including glucose 6-phosphate dehydrogenase (G6PD) [161].

LncRNA IDH1-AS1, a transcriptional target of c-Myc, promoted homodimerization of IDH1 and enhanced its enzymatic activity, which resulted in increased α-KG and decreased ROS production, leading to attenuation of glycolysis [162]. In addition, prostate cancer gene expression marker 1 (PCGEM1), an androgen-induced prostate-specific lncRNA, has been shown to promote glucose uptake for aerobic glycolysis, which is accompanied by the shunt of pentose phosphate to facilitate biosynthesis of nucleotides and lipids, and generate NADPH for redox homeostasis [163].

#### Regulation of amino acid metabolism by ROS and ncRNAs

As another important cellular energy source, amino acids also play a crucial role for the survival and proliferation of cancer cells. One crucial amino acid is glutamine which is converted into glutamate by the rate limiting enzyme glutaminase (GLS1/GLS2). It has been reported that overexpression of lncRNA urothelial carcinoma-associated 1 (UCA1) promoted the expression of GLS2 in human bladder cancer cells. Further study revealed that UCA1 functioned as a miR-16 sponge to reduce ROS production and induce GLS2 expression, leading to increased glutaminolysis in bladder cancer cells [164]. In a recent study, the authors found that miR-9-5p was significantly downregulated in pancreatic cancer tissues and cell lines. Overexpression of miR-9-5p inhibits the expression level of GOT1 mRNA by direct binding to its 3’UTR, thus affecting the glutamine metabolism and redox homeostasis in pancreatic cancer cells, suggesting that miR-9-5p may serve as a prognostic or therapeutic target for pancreatic cancer [165]. In MYC-driven liver tumors, the expression of GCLC, a rate-limiting enzyme of GSH synthesis, is attenuated by MYC-induced miR-18a expression, which contributes to GSH depletion and corresponds with increased sensitivity to oxidative stress in tumors [166]. Ferroptosis is a regulated form of cell death dependent on lipid-based ROS accumulation. It has been reported that miR-137 negatively regulates ferroptosis by directly targeting glutamine transporter SLC1A5 in melanoma cells, and knockdown of miR-137 increases the antitumor activity of erastin by enhancing lipid ROS-induced ferroptosis both in vitro and in vivo [167]. In gastric cancer cells, treatment of physcion 8-O-β-glucopyranoside, a common anthraquinone found in various plants, decreased expression of miR-103a-3p which released the expression of GLS2 and promoted ROS level and subsequent ferroptosis [168].

### ROS and ncRNAs in inducing angiogenesis

**Fig. 5:**
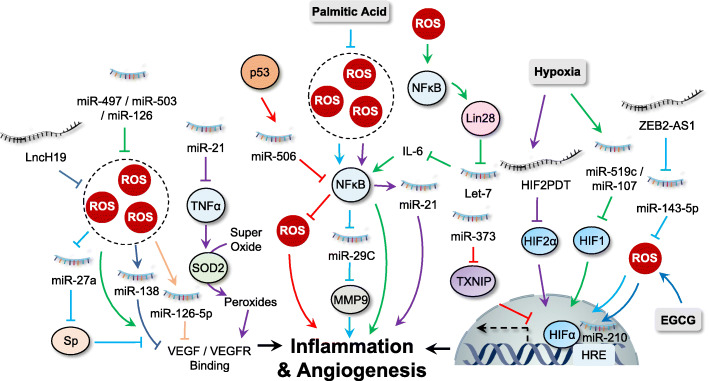
Interplay between ROS and ncRNAs in regulating inflammation and angiogenesis. ROS and ncRNAs regulate cancer inflammation and angiogenesis mainly by targeting NF-κB, HIF-1α and VEGFR signaling

When a tumor grows to a certain size, the primary proximal blood vessels are insufficient to support enough nutrients and oxygen [169]. Tumor cells then secret pro-angiogenic factors to stimulate new blood vessel formation, which is termed angiogenesis, an important hallmark of cancer [170]. The newly formed blood vessels provides oxygen and nutrition for tumor growth and remove metabolic waste from the tumor microenvironment [171]. Fibroblast growth factor 2 (FGF2), vascular endothelial growth factor (VEGF) and EGF are the three most potent pro-angiogenic factors in the vascularization of tumors [172]. In addition, the hypoxia inducible factor (HIF) complex is a major regulator of pro-angiogenic genes under low oxygen conditions [172]. Growing evidence has demonstrated the crucial role of the interaction between ROS and ncRNAs in inducing tumor angiogenesis (Fig. 5).

#### VEGF: a pivotal growth factor involved in angiogenesis regulated by ROS and ncRNAs

VEGF acts as an effector to stimulate cell proliferation and angiogenesis by binding to its transmembrane receptor, VEGFR. In NSCLC cells, ROS-induced overexpression of heme oxygenase-1 (HMOX1) contributed to p53-mediated inhibition of miR-378 expression, which reduced VEGF expression and diminished angiogenic potential [173]. A shortage of nutrition and oxygen in glioblastoma cell may lead to ROS accumulation. Under this stress condition, cancer cells displayed increased levels of miR-17 which targeted PTEN to upregulate VEGF to support cancer progression [174]. In addition, treatment of tubeimoside-1, a traditional Chinese herb, induced ROS accumulation and increased expression of miR-126-5p, which acted synergistically to target and downregulate VEGF-A and VEGF-R2, leading to growth inhibition of NSCLC cells [175]. Similarly, treatment with curcuminoids in colon cancer cells inhibited cancer progression by disrupting the ROS/miR-27a/Sp axis which mediated the inhibition of VEGF signaling [48]. ER stress-induced superoxides are converted into peroxides by TNF-α-mediated SOD activation. miR-21 has been reported to inhibit TNF-α, which prevents the conversion of superoxide to peroxide, leading to reduced binding of VEGF/FGF2 to their receptors [176]. In addition, there are several anti-angiogenic miRNAs, including miR-497, miR-503, and miR-126, which inhibit the ROS-mediated feedback loop of VEGFA/FGF2 [177–179]. Another report indicated that genotoxic stress-induced miR-494 expression suppressed DNA repair and angiogenesis through regulation of the MRE11a/RAD50/NBN (MRN) complex which positively correlated with VEGF signaling [180]. LncRNA H19 inhibits oxidative stress in cancer cells. Glioma cells show upregulation of H19, which act as a ceRNA for inhibition of miR-138, leading to activation of VEGF signaling and subsequent glioma angiogenesis [181]. Inhibition of lncRNA MALAT1 has been reported to increase cellular ROS levels. HCC cells upregulated MALAT1 to promote VEGF-A expression and angiogenesis via sponging miR-140 [182].

#### Regulation of HIF1-α by ROS and ncRNAs

Hypoxia is a key factor required for angiogenesis. It induces transcription factor HIF1 to activate the expression of pro-angiogenic factors, especially the VEGF family, thereby regulating tumor angiogenesis [183]. The HIF-1 complex is a heterodimeric protein consisting of two subunits, HIF-1α and HIF-1β. Under normoxia conditions, the stability of HIF-1α is accurately controlled by prolyl hydroxylase enzymes (PHDs)-mediated hydroxylation and subsequent proteasome degradation [171]. It has been reported that HIF-1α is stabilized by ROS, as Cys326 of PHD2 is oxidized by ROS leading to inactivated dimerization which results in HIF-1α accumulation and subsequent nuclear translocation [184].

In addition, growing data have suggested that HIF signaling is able to regulate cellular ROS levels, which may influence the transcription of several ncRNAs. For example, treatment with EGCG in lung cancer cells induces ROS accumulation which stabilizes HIF-1α. Stabilized HIF-1α thus binds to hypoxia response element (HRE) in proximity to miR-210 promoter, leading to overexpression of miR-210 and reduced cell proliferation [185]. Similarly, miR-224 was upregulated by hypoxia and HIF-1α in gastric cancer. Increased expression of miR-224 targeted Ras association domain family member 8 (RASSF8) to promote gastric cancer cell growth, migration and invasion, suggesting miR-224 as a potential therapeutic target for hypoxic gastric cancer patients [186]. HIF-1α was also found to repress miR-34a expression in p53-defective CRC cells, which activated STAT3 pathway and promoted EMT and metastasis [187]. In addition, the transcription of lncRNA HOTAIR is also regulated by HIF1α which binds to the HRE present in the HOTAIR promoter under hypoxia [188]. Similar findings are also reported in pancreatic cancer in which the transcription of lncRNA BX111 is induced by HIF-1α in response to hypoxia [189].

In contrast, ncRNAs are also found to be involved in the regulation of HIF-1α and related signaling [190]. Thioredoxin is known to reduce ROS levels in cancer cells, thereby controlling redox homeostasis. miR-373 has been found to bind to the 3’UTR of thioredoxin interacting protein (TXNIP) and downregulate its expression, which activates HIF-1α to promote cancer progression [191]. miR-497 regulates cisplatin chemosensitivity in cancer cells in a ROS-dependent manner. Wu and colleagues found that ectopic expression of miR-497 in breast cancer cells reduced HIF-1α and VEGF protein levels, thereby suppressing angiogenesis [192, 193]. The interaction between miR-21 and ROS has been reported in several cancer types. Studies have found that overexpression of miR-21 in Du145 human prostate cancer cells increased the expression of HIF-1α and VEGF, and induced tumor angiogenesis [194]. In gastric cancer cells, lncRNA zinc finger E-box-binding homeobox 2 antisense RNA 1 (ZEB2-AS1) was overexpressed and inhibited miR-143-5p expression by acting as a miRNA sponge, which increased ROS levels and HIF-1α expression to promote cancer progression [195]. The hypoxia-inducible factor-2α promoter upstream transcript (HIF2PUT) is a novel lncRNA which functions in angiogenesis by regulating the transcriptional activity of its host gene HIF-2α [131, 196]. Overexpression of HIF2PUT promotes HIF-2α expression to activate transcription of many pro-angiogenic factors under hypoxia conditions [197]. LncHIFCAR (long noncoding HIF-1α co-activating RNA) was also found to act as a HIF-1α co-activator which promoted HIF-1α activation and oral cancer progression [198].

### ROS and ncRNAs in tumor-promoting inflammation

Persistent chronic inflammation, either in tumor cells or the tumor microenvironment, has been recognized as a key regulator for tumorigenesis and been summarized as one of the cancer hallmarks [1]. Under chronic inflammation condition, ROS (or RNS) are generated not only from inflammatory cells but also epithelial cells, which may cause biomacromolecule damage and epigenetic alterations, thereby inducing cell transformation and malignancy [199]. The inflammatory processes regulate cancer progression based on the level of inflammation-related factors, cytokines, and chemokines produced from tumor cells or cells in the tumor microenvironment [9]. In addition, several transcriptional factors, including NF-κB, activator protein-1 (AP-1) and members of the STAT families, are involved in regulating inflammatory response by controlling the expression of inflammation-related factors [200]. Dysregulated expression of ncRNAs and their interplay with ROS have been linked to inflammation and tumorigenesis (Fig. 5).

#### NF-κB: the master regulator of inflammation which regulated by ROS and ncRNAs

The transcription factor NF-κB is probably the most well-known signaling factor in response to inflammation and is conserved in all multicellular animals [201]. NF-κB-regulated inflammation response may play a double-edged role in cancer progression. On the one hand, activation of NF-κB targets and eliminates transformed cells by inducing cytotoxic immune cells under acute inflammatory processes [202]. On the other hand, constitutive activation of NF-κB is commonly observed in many types of cancer which exerts a variety of pro-tumorigenic functions [201]. Accumulating evidence suggests that the interplay between ROS, ncRNA, and NF-κB contributes to tumorigenesis.

Several miRNAs have been shown to be transcriptional targets of NF-κB. For instance, it has been shown that saturated palmitic acid triggers generation of ROS and activation of NF-κB in pancreatic cancer cells. Furthermore, activation of NF-κB downregulates miR-29c, a negative regulator of extracellular matrix proteins MMP-9, thereby promoting pancreatic cancer progression [203]. Similarly, K-Ras signaling in pancreatic cancer activates NF-κB, which results in enhanced miR-155 expression, repressed FOXO3 expression and increased ROS accumulation [66]. In addition, increased expression of miR-21 is a downstream event of ROS-mediated NF-κB activation and exhibits a crucial role in arsenic-induced neoplastic transformation in human lung embryo fibroblast cells [204]. Similarly, treatment of human multiple myeloma cell line U266 with berberine resulted in Set9-mediated lysine methylation of the RelA subunit, which inhibited NF-κB nuclear translocation and miR-21 transcription, thereby inducing ROS generation and growth suppression [205]. Furthermore, NF-κB also promotes the expression of proteins that regulate miRNAs. The most important example is the NF-κB-dependent induction of lin28, whose expression inhibits the maturation of let-7 miRNAs. As IL-6 is one of the targets of let-7 miRNAs, the reduced expression of let-7 leads to higher levels of IL-6 and further activation of NF-κB, therefore generating a positive feedback loop [206].

In addition to regulating miRNAs directly or indirectly, NF-κB activity itself is regulated by several miRNAs. For example, p53-mediated miR-506 overexpression induces ROS accumulation via negative regulation of NF-κB (p65), thereby exhibiting a tumor suppressive role in lung cancer cells [207]. Upregulation of miR-223 promoted mitochondria ROS production and suppressed the phosphorylation of RelA, which inhibited NF-κB-regulated transcription of pro-inflammatory genes, including IL-1β, IL-6, TNF-α, and IL-12p40 [135, 208]. miR-9 overexpression promotes ROS production in multiple myeloma, which targets TRIM56 and activates the NF-κB pathway to promote the development and progression of multiple myeloma [209]. In addition, ROS-mediated upregulation of miR-124-3p specially binds to the 3’UTR region of neuropilin-1 and suppress its expression, which negatively regulates PI3K/Akt/NF-κB pathways, leading to growth suppression in GBM cells [210, 211]. miR-17-92 has been found to decrease ROS levels in cancer cells. Overexpression of the miR-17-92 targets tumor necrosis factor receptor associated factor 3 (TRAF3) and release its inhibition on the NF-κB signaling pathway, thus promoting gastric cancer progression [71, 212].

#### Regulation of AP-1/STAT: essential transcription factors for inflammatory response

The activation of the transcription factor AP-1 and members of the STAT families are essential for maintaining cellular homeostasis under inflammatory conditions [213]. Dysregulation of JAK/STAT signaling has been implicated in the regulation of inflammatory response in malignant cells and ncRNAs have been demonstrated to target important players in this pathway. For example, overexpression of miR-124 was found to inhibit the expression of SIRT1 and thus promoted the generation of ROS, which induced miR-124 binding to the 3’UTR region of STAT3 and inhibited the expression of STAT3 proteins, resulting in reduced cell proliferation in HCC cells [214, 215]. In addition, overexpression of miR-33a was observed in clinical glioma specimens and cell lines, which negatively regulated the expression of SIRT6 by targeting its mRNA 3’UTR. Further study found that overexpression of SIRT6 reduced cell survival and initiated apoptosis by enhancing ROS level and inhibiting the JAK3/STAT3 pathway [101]. The lncRNA UCA1 reduced ROS production in cancer cells. It has been reported that UCA1 acts as miRNA sponge to inhibit miR-126 expression, thus activating JAK/STAT signaling pathways and promoting cell proliferation in human leukemia cells [216]. Moreover, treatment with aspirin promotes ROS production and activates lncRNA OLA1P2 expression. OLA1P2 upregulation markedly inhibits the nuclear transport of phosphorylated STAT3 by binding to and preventing homodimerization of phosphorylated STAT3, thus inhibiting STAT3 signaling and suppressing cancer progression [217].

### ROS and ncRNAs in invasion and metastasis

**Fig. 6:**
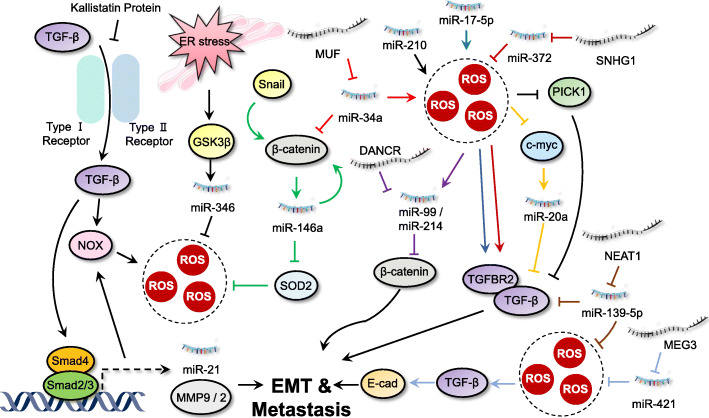
Interplay between ROS and ncRNAs in regulating invasion and metastasis. The interaction between ncRNAs and ROS indicates a crucial role for both positive or negative regulation of Wnt and TGF-β pathways to modulate invasion and metastasis of cancer cells

Metastasis is a complex and multifaceted event which involves the process of invasion, intravasation into blood, extravasation to distant organs and growth [218]. A prerequisite change before metastasis is the activation of epithelial-mesenchymal transition (EMT) in cancer cells, a phenotypic transition of epithelial cells to acquire more aggressive mesenchymal characteristics [218]. In addition, colonization of metastatic cells is another important process, which allows the formation of metastatic niches in distant organs [219]. The multistep process of metastasis is regulated by several transcriptional factors such as Snail, Slug, ETS-1, Twist, ZEB1 and ZEB2 [220–223]. Moreover, several targets of these transcriptional factors are closely related to the metastatic process, such as metalloproteases (MMP-2/9) and chemokines or cytokines like transforming growth factor beta (TGF-β) [224, 225]. Along with protein-coding genes, some ncRNAs may participate in cancer metastasis and the interplay between ROS and ncRNAs in cancer metastasis has been documented (Fig. 6).

#### Regulation of TGF-β signaling by ROS and ncRNAs

TGF-β, an important pleiotropic cytokine, regulates cell proliferation, cell adhesion, cell migration, and the differentiation of a plethora of different cell types [226]. Generally, canonical TGF-β signaling promotes the expression of mesenchymal markers (such as N-cadherin and vimentin) and reduces the epithelial markers (like E-cadherin) via transcription factor SMAD family proteins [227]. TGF-β signaling displays a double-edged role in regulation tumorigenesis. It acts as a tumor suppressor at early stages of cancer by inducing cytostasis and apoptosis. However, at later stages, it functions as an oncoprotein by supporting cancer growth and inducing metastasis, thereby promoting the development and progression of cancer [226].

TGF signaling has been reported to closely interact with ROS and ncRNA in regulating cancer metastasis. For example, upregulation of miR-200 family is positively correlated with increased ROS levels upon chemotherapy or radiotherpy in cancer cells [228]. TGF-β has been found to downregulate miR-200 family members, including miR-200a/−200b/−200c/− 141/− 429, which promote ZEB1 and ZEB2 expression and subsequent cancer progression [229]. TGF signaling and ROS upregulate miR-182 expression, which sustains NF-κB activation by directly suppressing cylindromatosis (CYLD, an NF-κB negative regulator) [230]. Overexpression of miR-182 also reduce SMAD7 expression and promote breast cancer invasion and TGF-β-induced bone metastasis [231]. TGF signaling and ROS also induce the expression and promoter activity of miR-155, which reduces RHOA expression and disrupts tight junctions, leading to invasion and metastasis of breast cancer [232]. The expression of miR-206 is regulated by Nrf2 under oxidative stress conditions [155]. miR-206 has been found to inhibit autocrine production of TGF-β and downstream neuropilin-1 (NRP1) and SMAD2 expression, leading to decreased migration, invasion, and EMT in breast cancer cells [233]. In primary myelofibrosis CD34+ cells, TGF-β signaling enhances miR-382-5p expression and reduces its target gene SOD2 activity, leading to ROS overproduction [234]. Guo et al. demonstrated that the kallistatin protein (a plasma protein) inhibited TGF-β signaling and ROS production via its heparin-binding site, which further blocked miR-21 and AKT signaling, thus suppressing EMT [235].

In another study, metformin-induced SOD overexpression quenched ROS levels and blocked the TGF pathway, which inhibited miR-21 and MMP-2/9 expression, leading to suppression of cell proliferation and/or migration [106]. In irradiated NSCLC cells, accumulation of ROS results in the activation of TGF signaling and subsequent overexpression of miR-21, which leads to DNA damage [236]. ROS-induced decrease in c-Myc expression downregulates miR-20a [237]. TGF-β and one of its receptors (TGFBR2) were found to be downregulated by miR-20a via direct binding of miR-20a to its 3’UTR, thus abrogating the TGF-β signaling in breast cancer cells [238]. Overexpression of miR-210 results in an increase of ROS generation in cancer cells [239]. Upregulated miR-210 targets protein interaction with PRKCA 1 (PICK1), a critical negative regulator of the TGF-β pathway, and inhibits its expression. This activates TGF-β signaling and promotes bone metastasis of prostate cancer [240]. miR-17-5p has been found to increase ROS levels by inhibiting three major antioxidant enzymes, MnSOD, GPX2, and TrxR2 [47]. A luciferase reporter assay identified TGFBR2 as its target and overexpression of miR-17-5p significantly enhanced cervical cancer cell proliferation and metastasis [241]. Moreover, miR15a/16 has been found to induce mitochondrial ROS production and reduce the expression of endogenous Smad3, thereby inhibiting invasion of prostate cancer cells by suppressing the TGF-β signaling pathway [44, 242].

In addition to miRNAs, ROS and lncRNAs are also involved in regulation of TGF-β signaling during cancer progression. Overexpression of miR-139-5p significantly increased oxidative stress via targeting ROS defense pathways [243]. LncRNA NEAT1 functions as a ceRNA by sponging miR-139-5p, which upregulates TGF-β1 to promote HCC progression [244]. Inhibition of lncRNA MALAT1 has been reported to increase cellular ROS levels. Upregulated TGF-β in head and neck squamous cell carcinoma (HNSCC) may promote STAT3 activation, which binds to the MALAT1 promoter and activates its expression, thereby inducing EMT and accelerating HNSCC metastasis [245]. The expression of miR-1 is regulated by Nrf2 [155]. LncRNA UCA1 functions as a ceRNA for titrating miR-1 and miR-203a to increase Slug expression, which promotes TGF-β-induced EMT and invasion in metastatic breast cancer [246]. miR-421 decreases ROS levels by targeting KEAP1 expression [247]. It has been found that lncRNA MEG3 functions as a sponge of miR-421 to regulate E-cadherin expression, thereby promoting TGF-β-induced EMT in breast cancer [248]. miR-372 has been found to decrease ROS levels by targeting p62 in cancer cells [249]. LncRNA lnc-SNHG1 significantly promotes the expression of TGFBR2 and RAB11A via sponging miR-302/372/373/520, thus activating EMT in invasive pituitary cancer [250]. Another study found that lncRNA XIST functions as an ceRNA to inhibit miR-137 expression and decrease ROS levels simultaneously, which promote ZEB2 expression and subsequent TGF-β-induced EMT in NSCLC cells [251, 252].

#### Regulation of Wnt/β-catenin signaling: a well-known regulator for tumor metastasis

Wnt/β-catenin signaling, an evolutionarily highly conserved pathway, controls a multitude of developmental processes, including embryonic development and maintenance of adult tissue homeostasis [253]. Wnt signaling is stage-specific or cancer type-specific and functions by regulating the expression of specific target genes, such as c-myc, E-cadherin, and cyclin D1 [254, 255]. NcRNAs have been found to contribute to both the activation and inactivation of Wnt signaling for regulating tumorigenesis. It has been shown that ER stress can upregulate miR-346 expression, which reduces ROS levels through mitophagy [256]. ER stress also enhances glycogen synthase kinase-3 beta (GSK3β) expression, and GSK3β inhibition reverses the effects of miR-346 on ROS production, suggesting the role of Wnt signaling in regulating cancer progression through the miR-346/ROS/GSK3β axis [256]. miR-146a downregulates the expression of SOD2 and enhances ROS generation in cancer cells [257]. It has been reported that Snail can induce the expression of miR-146a through β-catenin/TCF4, which in turn stabilizes β-catenin and forms a positive feedback circuit, leading to sustained activation of Wnt signaling in CRC stem cells [258]. Generation of ROS activates miR-199/214 transcription. The lncRNA DANCR binds to the 3′TUR of CTNNB1 mRNA and blocks the inhibitory effect of miR-214 and miR-199, which in turn increases CTNNB1 protein levels and promotes subsequent activation of Wnt signaling in HCC cells [259]. In addition, miR-34a targets NOX2 to enhance ROS production [260]. The lncRNA-MUF (mesenchymal stem cell-upregulated factor) can act as a ceRNA for miR-34a and promote EMT by upregulating Snail1 expression and activating Wnt/β-catenin signaling [261].

In addition to the above signaling events, there exist several other metastasis-associated factors that interact with ncRNAs in controlling cancer progression. miR-21, a well-known oncogenic microRNA contributing to carcinogenesis in prostate and other cancers, was upregulated by ROS-mediated Akt activation, which contributed to the highly invasive and metastatic phenotype of prostate cancer cells by downregulation of maspin and PDCD4 [262]. Secreted protein acidic and rich in cysteine (SPARC) is a matrix protein which mediates diverse cellular functions and has an important role in regulation of cell-matrix interactions and migration. An oncogenic microRNA miR-155 has been reported to decrease the expression of tumor protein 53 induced nuclear protein 1 (TP53INP1), a p53 target gene responsible for the p53-driven oxidative stress response, thereby upregulating SPARC expression and promoting subsequent cell migration in pancreatic adenocarcinoma [263]. lncRNA H19 and HULC were upregulated by oxidative stress and regulated cholangiocarcinoma cell migration and invasion by targeting IL-6 and CXCR4 via ceRNA patterns, which sponge let-7a/let-7b and miR-372/miR-373, respectively [264].

### ROS and other types of ncRNAs in regulating cancer hallmarks

Although miRNAs and lncRNAs are the main types of ncRNAs interacting with oxidative stress to regulate cancer hallmarks, other ncRNAs are also found to interact with oxidative stress for regulation of cancer progression, such as circRNAs. As the unique structure of circRNAs that contain covalently joined 3′ and 5′ ends, it is rational to hypothesize that circRNAs regulate cancer progression by acting as miRNA sponges [265]. Indeed, circRNAs are implicated in a variety of cancers (including gastric cancer, hepatocellular cancer, bladder cancer, and esophageal cancer, and others) [266, 267]. For example, knockdown of circular ATP binding cassette subfamily B member 10 (circABCB10) promoted lipid ROS production and subsequent ferroptosis by regulating the miR-326/CCL5 axis in rectal cancer, indicating circABCB10 as a promising therapeutic target for rectal cancer [268]. Similarly, circ-TTBK2 was upregulated in glioma tissues and cells. Further study found that circ-TTBK2 was a sponge of miR-761 to modulate ITGB8, and knockdown of circ-TTBK2 induced lipid ROS production, which promoted ferroptosis and retarded cell proliferation, invasion in glioma cells, suggesting a potential biomarker for clinical glioma treatment [269]. The folate cycle plays a key role in the production of NADPH for neutralization of ROS. Circ_0062019 and its host gene SLC19A1 were significantly upregulated in prostate cancer. Upregulated SLC19A1 thus encode a membrane protein to transport folate, which decreases ROS levels and promotes prostate cancer proliferation [270]. Therefore, these above findings demonstrate that the interplay between circRNAs and oxidative stress plays dominant role in regulating cancer hallmarks. And more types of ncRNAs will be identified to interact with oxidative stress for regulating cancer hallmarks in the future.

## Conclusions

To date, mounting evidence has demonstrated the critical role of ROS in tumor progression, which display a double-edged function by acting either as a second messenger for signal transduction or damaging macromolecules/organelles for cell death. During these processes, ROS can modulate biological function by regulating several protein-coding genes (either onco- or tumor suppressive) involved in most hallmarks of cancer, thereby regulating cancer progression. In this review, we highlight that ROS also function in cellular biological events by interaction with non-coding transcripts, especially miRNAs and lncRNAs. Indeed, the interplay between ROS and miRNAs/lncRNAs is tightly associated with diverse biological processes, including cancer cell proliferation, cell senescence and cell invasion, suggesting their crucial role in the regulation of most cancer hallmarks. In summary, the interplay between ncRNAs and ROS holds great potential for the development of novel detection biomarkers or therapeutic strategies for future cancer treatment.

## Prospective

The interaction between ncRNAs and ROS takes place either during ncRNA biogenesis, at the epigenetic level or during the signal transduction. In addition, most studies indicate the interplay between ROS and ncRNAs are miRNAs-based. Studies exploring the association with lncRNA/circRNAs and ROS are still relatively limited, although lncRNA and circRNA may play more important roles during cancer progression. As circRNAs are abundant, conserved and stable in mammalian cells, these advantages make it relatively easy to detect and hold the potential to be effective cancer biomarker in the future. Thus, the possible future research focuses should pay more attention to the regulatory role of circRNA in cancer.

Moreover, most ncRNAs studied to date interact with ROS to regulate cell growth-related cancer hallmarks, such as sustaining proliferative signaling, evading growth suppression, and resisting cell death. However, only limited data exists regarding regulation of evading immune destruction of cancer cells. Indeed, immunotherapy is now one of the most effective treatment strategies for several types of cancer. ROS and ncRNAs alone have been demonstrated to regulate immune escape in cancer treatment, but researches referring to the interplay between ROS and ncRNAs in immune escape are limited. Further studies are therefore needed for deeper understanding their regulatory roles in cancer immune escape.

Additionally, studies have found that several ncRNAs interact with ROS to participate in regulation of more than one cancer hallmark. Taking miR-21 as an example, it is able to be regulated by or regulate ROS generation in cancer cells and modulate several cancer hallmarks, including sustaining proliferative signaling, evading growth suppression, resisting cell death, inducing angiogenesis, tumor-promoting inflammation, and invasion and metastasis. This may be an important issue which should be addressed when targeting the interplay between ncRNAs and ROS for cancer therapy due to the complex interaction patterns and diverse molecular mechanisms involved. The poor pharmacokinetics of ncRNAs are also considered as an obstacle of ncRNA-based targeted therapy. To date, most of the studies related to ncRNAs and ROS are conducted in in vitro cell lines, which herald delays in the timeline for implication of ncRNA-based targeted therapy in cancer treatment.

Nevertheless, with the technical advancements in cellular and molecular biology coupled with bioinformatic approaches, almost certainly more and more ncRNAs will be found to work together with ROS to regulate the hallmarks of cancer. Moreover, increased use of cancer animal models in future studies will pave the way for translation of in vitro results into clinical applications.

## Data Availability

All the data obtained and/or analyzed during the current study were available from the corresponding authors on reasonable request.

## Abbreviations

lncRNA: long noncoding RNA
ncRNA: noncoding RNA
ROS: Reactive oxygen species
H_2_O_2_: Hydrogen peroxide
miRNA: microRNA
piRNA: PIWI-interacting RNA
snoRNA: small nucleolar RNA
siRNA: small interfering RNA
MREs: miRNA response elements
lincRNA: intergenic lncRNA
elncRNA: enhancer lncRNA
GAS5: growth arrest specific 5
MALAT1: Lung adenocarcinoma transcript-1
circRNA: circular ncRNA
ceRNA: competitive endogenous RNA
ER: Endoplasmic reticulum
PKM2: Pyruvate kinase M2
RTK: Receptor tyrosine kinases
CDK: Cyclin-dependent kinase
CDKI: CDK inhibitor
IR: Ionizing radiation
ALA-SDT: 5-aminolevulinic acid-mediated sonodynamic therapy
MnSOD: Manganese superoxide dismutase
HCC: Hepatocellular carcinoma
ASO: Antisense oligonucleotide
EGFR: Epidermal growth factor receptor
Sp: Specificity protein
MAPK: Mitogen-activated protein kinases
ESCC: Esophageal squamous cell carcinoma
RB: Retinoblastoma
FOXO: Forkhead box O
TNBC: Triple-negative breast cancer
PLC: Peptide-loading complex
HDACi: Histone deacetylase inhibitor
UTR: Untranslated region
BA: Betulinic acid
POX: Proline oxidase
NEAT1: Nuclear enriched abundant transcript 1
EGCG: Epigallocatechin-3-gallate
CTR1: Copper transporter 1
Bcl-2: B-cell lymphoma 2
PDCD4: Programmed cell death 4
Keap1: Kelch-like ECH-associated protein 1
ARE: Antioxidant-response-element
EpRE: Electrophile-response element
PRXL2A: Peroxiredoxin-like 2A
OSCC: Oral squamous cell carcinoma
SCAL1: Smoke- and cancer-associated lncRNA-1
TUG2: Taurine-upregulated gene 2
KRAL: Keap1 regulation-associated lncRNA
CSC: Cancer stem cell
GSC: Glioma stem cell
TNBCSC: Triple-negative breast cancer stem cell
HAX-1: Hematopoietic cell-specific protein 1-associated protein X-1
OXPHOS: Oxidative phosphorylation
PDAC: Pancreatic ductal adenocarcinoma
PFK1: Phosphofructokinase 1
TCA: Tricarboxylic acid
PDK2: Pyruvate dehydrogenase kinase 2
PDH: Pyruvate dehydrogenase
GLUT1: Glucose transporter-1
HK2: Hexokinase 2
PTBP1: Polypyrimidine tract-binding protein 1
PPP: Pentose phosphate pathway
G6PD: Glucose 6-phosphate dehydrogenase
PCGEM1: Prostate cancer gene expression marker 1
UCA1: Urothelial carcinoma-associated 1
FGF2: Fibroblast growth factor 2
VEGF: vascular endothelial growth factor
HIF: Hypoxia inducible factor
HMOX1: Heme oxygenase-1
PHD: Prolyl hydroxylase enzymes
HRE: Hypoxia response element
TXNIP: Thioredoxin interacting protein
ZEB2-AS1: Zinc finger E-box-binding homeobox 2 antisense RNA 1
HIF2PUT: Hypoxia-inducible factor-2α promoter upstream transcript
AP-1: Activator protein-1
TRAF3: Tumor necrosis factor receptor associated factor 3
EMT: Epithelial-mesenchymal transition
NRP1: Neuropilin-1
PICK1: Protein interaction with PRKCA 1
HNSCC: Head and neck squamous cell carcinoma
GSK3β: Glycogen synthase kinase-3 beta
MUF: Mesenchymal stem cell-upregulated factor
SPARC: Secreted protein acidic and rich in cysteine
TP53INP1: Tumor protein 53 induced nuclear protein 1
